# COX inhibition reduces vasodilator PGE_2_ but is shown to increase levels of chemoattractant 12‐HETE *in vivo* in human sunburn

**DOI:** 10.1111/exd.12734

**Published:** 2015-06-03

**Authors:** Suzanne M. Pilkington, Sharon A. Murphy, Shobha Kudva, Anna Nicolaou, Lesley E. Rhodes

**Affiliations:** ^1^ Centre for DermatologyInstitute of Inflammation and RepairUniversity of ManchesterSalford Royal NHS Foundation TrustManchester Academic Health Science CentreManchesterUK; ^2^ Manchester Pharmacy SchoolFaculty of Medical and Human SciencesUniversity of ManchesterManchesterUK

**Keywords:** 12‐hydroxyeicosatetraenoic acid, 12‐lipoxygenase, cyclooxygenase prostaglandin, E2, ultraviolet radiation

**Abbreviations**
COX: cyclooxygenase
12‐HETE: 12‐hydroxyeicosatetraenoic acid
12‐LOX: 12‐lipoxygenase
PGE_2_: prostaglandin E_2_
UVR: ultraviolet radiation

## Conflict of interests

The authors have declared no conflicting interests.

To the Editor,

Ultraviolet radiation (UVR) induces acute skin inflammation, characterized clinically by erythema, histologically by dermal leukocytic infiltration and biochemically by upregulation of pro‐inflammatory eicosanoids 1. Metabolism of arachidonic acid (AA) released from membrane lipids enhances eicosanoid production, with notable elevation of prostaglandin (PG)E_2_ and 12‐hydroxyeicosatetraenoic acid (12‐HETE), major metabolites of cyclooxygenase (COX) and 12‐lipoxygenase (12‐LOX) pathways, respectively. At low concentrations, PGE_2_ has roles in skin homeostasis, while UVR‐upregulated PGE_2_ has a significant vasodilatory effect in the erythemal response 2. A 12‐fold increase in 12‐HETE occurs in UVR‐inflamed skin 1, and this monohydroxy fatty acid has potent leucocyte chemoattractant properties *ex vivo* 3, 4 and in animal models 5. Its role as a chemoattractant for neutrophils and lymphocytes in human skin is supported by their pronounced infiltration following intradermal injection of 12‐HETE 6; 12‐HETE may also increase leucocytic infiltration in lesional psoriatic skin 3, 4.

**Figure 1:**
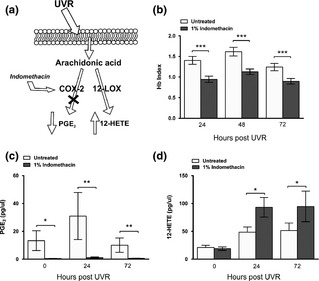
(a) Inhibition of UVR‐upregulation of COX‐2 activity may increase flux of available free AA through the 12‐ LOX pathway. (b–d) Impact of topical indomethacin treatment on UVR‐inflammation over a 72 h time‐course in human skin: (b) Erythema assessed as Hb Index using spectrophotometry (mean (SEM), *n* = 13), (c) levels of suction blister fluid PGE2 (mean (SEM), *n* = 5) and (d) 12‐HETE (mean (SEM), *n* = 10) measured by LC‐MS/MS.*P < 0.05, **P < 0.01, ***P < 0.001 compared to untreated skin.

Cyclooxygenase inhibitors are widely employed to treat inflammatory conditions including sunburn. Their therapeutic properties are principally attributed to reduction of PGE_2_ synthesis from AA 2. However, while COX inhibition reduces PGE_2_ concentration and erythema in UVR‐inflamed skin 2, reduced metabolism of AA via COX may potentially result in an enhanced availability of AA for LOX metabolism 7, paradoxically increasing aspects of UVR inflammation through augmentation of chemoattractant 12‐HETE (Fig. 1a). Accordingly, we have examined the impact of a COX inhibitor on PGE_2_ and 12‐HETE levels, and clinical and histological outcomes, in UVR‐induced inflammation in human skin.

Ethical approval was granted (Greater Manchester North NREC; reference 11/NW/0567) and the study was performed in accordance with Declaration of Helsinki principles (Seoul 2008); all volunteers gave written informed consent. Overall, the study involved 13 healthy individuals (phototype II/III; 20‐39 years; 8 female, 5 male). Initially, in *n* = 10, one‐cm‐diameter sites of previously photoprotected buttock skin were exposed to 3× each individual's minimal erythemal dose (MED) of UVR (TL 20W/12 lamp, Philips GmbH, Hamburg, Germany; 270–400 nm) and indomethacin gel (1%; Indomet‐ratiopharm^®^ Gel, Ratiopharm GmbH, Germany) was applied to UVR‐exposed and unexposed skin for 3 h under Tegaderm^™^ dressing on three occasions, immediately post‐UVR, after 24 and 48 h. Changes in haemoglobin (Hb) index were measured via reflectance spectrophotometry (CM‐600d, Konica Minolta Sensing Europe B.V.), at indomethacin‐treated and control untreated sites, in both UVR‐exposed and unexposed skin at 24, 48 and 72 h post‐UVR, prior to suction blister fluid sampling. Eicosanoid levels were analysed by LC‐MS/MS (described in Ref. 8, 9).

Topical indomethacin significantly reduced Hb index post‐UVR compared to untreated skin at 24, 48 and 72 h (all *p *<* *0.001; Fig. 1b). Skin PGE_2_ level (*n* = 5 for technical reasons) was almost completely abolished after indomethacin treatment in unexposed skin (mean 97% reduction; *p *<* *0.05) and at 24 h (96% reduction, *p *<* *0.01) and 72 h post‐UVR (94% reduction, *p *<* *0.01) (Fig. 1c). In contrast, levels of 12‐HETE (*n* = 10) increased substantially with indomethacin application, at both 24 h (92% increase) and 72 h (74% increase) post‐UVR compared to untreated skin (both *p *<* *0.05); no significant increase was seen in unexposed skin (Fig. 1d). While an increase in 12‐HETE production has been noted following COX inhibitor application *in vitro* (s1) including in human psoriatic epidermal extracts (s2), to our knowledge we present the first report of elevated 12‐HETE levels in skin *in vivo* resulting from COX inhibition.

In the light of the significantly increased 12‐HETE, we assessed the impact of topical indomethacin on the UVR‐induced inflammatory cell infiltrate. Thus, *n* = 4 volunteers (including one who participated in the blister study) underwent the same protocol as above, but provided skin punch biopsies (5 mm) from UVR‐exposed and unexposed skin at 24 and 72 h post‐UVR, at both indomethacin‐treated and untreated sites. Immunohistochemistry was performed on frozen skin sections (5 *μ*m) using mouse primary antibodies for neutrophil elastase (Dako, Cambridgeshire, UK), CD3 (Vector, Peterborough, UK) and CD8 (Dako). The UVR‐induced dermal neutrophil infiltration appeared increased after indomethacin versus untreated skin at 24 h (mean 48% increase) and 72 h (44% increase) post‐UVR (Fig. S1). Dermal T lymphocyte infiltration also appeared increased in indomethacin‐treated versus untreated skin, at 72 h CD3+ cells increased by 34% (Fig. S2) and CD8+ cells by 57% (Fig. S3). Subjects showed different time‐points (24/72 h) of maximum T‐cell infiltration; at their time‐point of maximum infiltration, the difference in CD8+ counts was statistically significant, mean 46.2 (SEM 10.32) versus 15.04 (3.9) in indomethacin‐treated vs untreated skin, respectively (*p *<* *0.05).

Thus, while COX inhibition reduces the erythemal component of sunburn, this occurs concurrent with higher cutaneous levels of chemoattractant 12‐HETE, alongside evidence of augmentation of the UVR‐induced inflammatory cell infiltrate. As skin COX‐2, in contrast to COX‐1 (s3), is strongly upregulated by UVR, these effects are anticipated to be largely due to COX‐2 inhibition. Increased synthesis of LOX‐produced mediators, such as 12‐HETE, could potentially contribute to adverse effects of COX inhibitors, as proposed for NSAID exacerbation of psoriasis (s4) and of asthma by aspirin (s5). Studies in mice also implicate the 12‐LOX pathway in pathogenesis of atherosclerosis and vascular inflammatory reactions in diabetes (s6, s7). There are potential further complexities surrounding the impact of NSAIDs, which in the sunburn response may include effect of the PGE_2_ reduction on immunosuppressive cytokine IL‐10 levels (s8) and on reparative activities of the response. Future research could explore these, as well as examine impact on histological findings in a larger group and potentially also in polymorphic light eruption patients, where dysregulated leucocytic infiltration is reported (s9).

Our study provides human *in vivo* data showing significant rise in skin chemoattractant 12‐HETE following application of a COX inhibitor, supporting a speculative role of the 12‐LOX pathway in adverse reactions associated with therapeutic COX inhibition. We highlight that indomethacin treatment of sunburn may be limited in its scope and that changes in UVR‐erythema do not, as commonly assumed, necessarily reflect histological leucocytic infiltration in this acute inflammatory response.

## Supporting information


**Figure S1** Impact of topical indomethacin on cutaneous neutrophil infiltration over a 72 h time‐course of UVR‐inflammation. (a) Neutrophil counts per high power field (HPF) (mean (SEM), n = 4) and (b) photomicrographs of sections showing neutrophil infiltration from indomethacin treated and untreated skin (scale bar 100μm).

**Figure S2** Impact of topical indomethacin on cutaneous CD3+ T cell infiltration over a 72 h time‐course of UVR‐inflammation. (a) CD3+ T cell counts per high power field (HPF) (mean (SEM), n = 4) and (b) photomicrographs of sections showing CD3+ T cell infiltration from indomethacin treated and untreated skin (scale bar 100μm).

**Figure S3** Impact of topical indomethacin on cutaneous CD8+ T cell infiltration over a 72 h time‐course of UVR‐inflammation. (a) CD8+ T cell counts per high power field (HPF) (mean (SEM), n = 4) and (b) photomicrographs of sections showing CD3+ T cell infiltration from indomethacin treated and untreated skin (scale bar 100μm).

**Appendix S1** References.
