# Supplementary figures and images for: COX inhibition reduces vasodilator PGE2 but is shown to increase levels of chemoattractant 12‐HETE in vivo in human sunburn
Source: Exp Dermatol. 2015 Jun 3;24(10):790–1. doi: 10.1111/exd.12734 (PMC4737235; doi:10.1111/exd.12734)

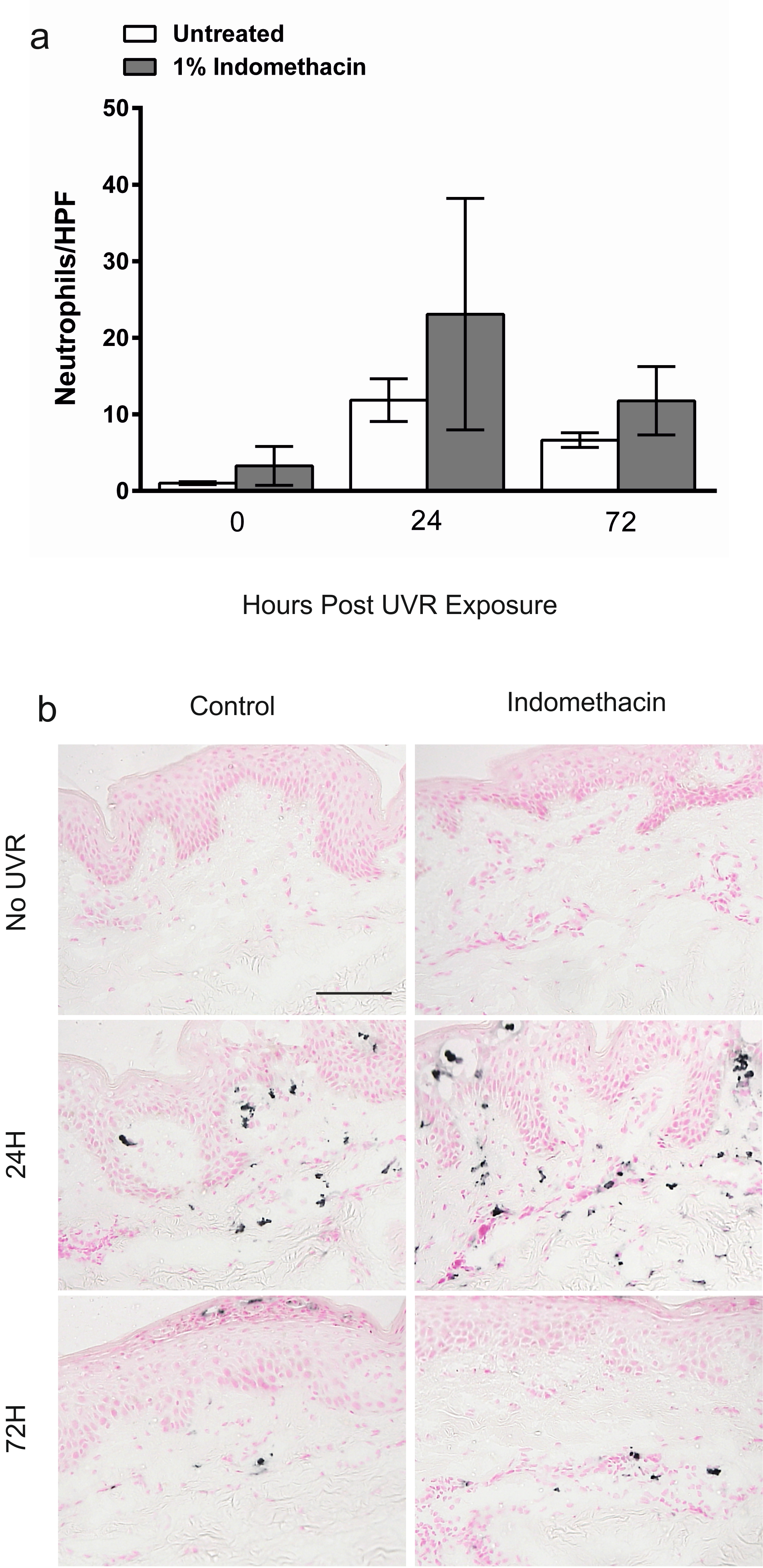

Supplement: Supplementary file 1 — Figure S1 Impact of topical indomethacin on cutaneous neutrophil infiltration over a 72 h time‐course of UVR‐inflammation. (a) Neutrophil counts per high power field (HPF) (mean (SEM), n = 4) and (b) photomicrographs of sections showing neutrophil infiltration from indomethacin treated and untreated skin (scale bar 100μm). [file EXD-24-790-s001.tif]

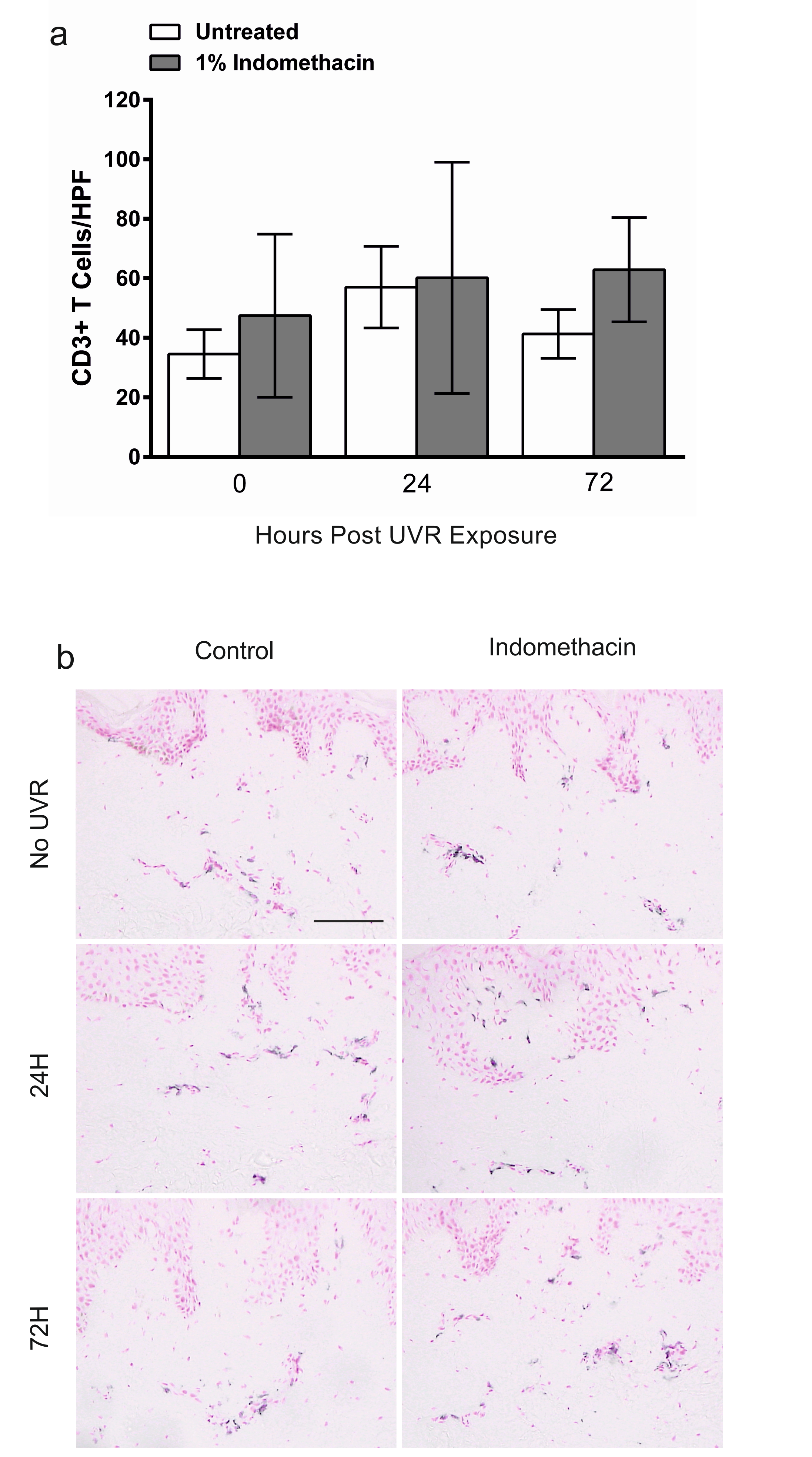

Supplement: Supplementary file 2 — Figure S2 Impact of topical indomethacin on cutaneous CD3+ T cell infiltration over a 72 h time‐course of UVR‐inflammation. (a) CD3+ T cell counts per high power field (HPF) (mean (SEM), n = 4) and (b) photomicrographs of sections showing CD3+ T cell infiltration from indomethacin treated and untreated skin (scale bar 100μm). [file EXD-24-790-s002.tif]

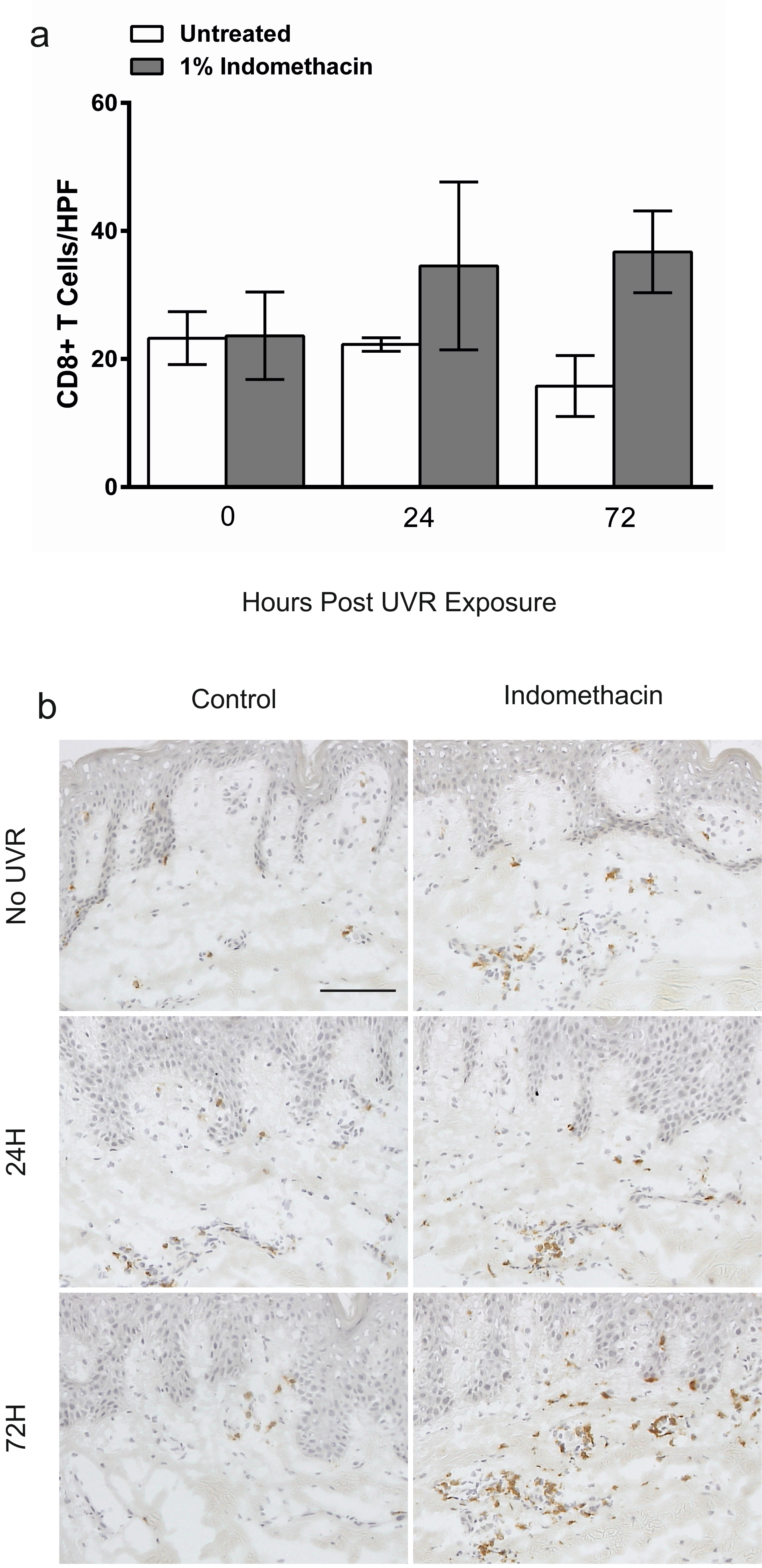

Supplement: Supplementary file 3 — Figure S3 Impact of topical indomethacin on cutaneous CD8+ T cell infiltration over a 72 h time‐course of UVR‐inflammation. (a) CD8+ T cell counts per high power field (HPF) (mean (SEM), n = 4) and (b) photomicrographs of sections showing CD3+ T cell infiltration from indomethacin treated and untreated skin (scale bar 100μm). [file EXD-24-790-s003.tif]
