# Supplementary material for: COX inhibition reduces vasodilator PGE2 but is shown to increase levels of chemoattractant 12‐HETE in vivo in human sunburn
Source: Exp Dermatol. 2015 Jun 3;24(10):790–1. doi: 10.1111/exd.12734 (PMC4737235; doi:10.1111/exd.12734)
Supplement: Supplementary file 4 — Appendix S1 References. [file EXD-24-790-s004.docx]

**Supplementary References**

s1. He C, Y Wu, Y Lai *et al.* Dynamic eicosanoid responses upon different inhibitor and combination treatments on the arachidonic acid metabolic network*.* Mol BioSyst 2012: 8: 1585-1594.

s2. Hammarstrom S, M Hamberg, B Samuelsson *et al.* Increased concentrations of nonesterified arachidonic acid, 12L-hydroxy-5,8,10,14-eicosatetraenoic acid, prostaglandin E2, and prostaglandin F2alpha in epidermis of psoriasis*.* Proc Natl Acad Sci U S A 1975: 72: 5130-5134.

s3. Seo J Y, E K Kim, S H Lee *et al.* Enhanced expression of cylooxygenase-2 by UV in aged human skin in vivo*.* Mech Ageing Dev 2003: 124: 903-910.

s4. Powles A V, C E Griffiths, M H Seifert *et al.* Exacerbation of psoriasis by indomethacin*.* Br J Dermatol 1987: 117: 799-800.

s5. Neighbour H Mechanisms of Aspirin-Intolerant Asthma: Identifying Inflammatory Pathways in the Pathogenesis of Asthma*.* Int Arch Allergy Immunol 2014: 163: 1-2.

S6. Cyrus T, J L Witztum, D J Rader *et al.* Disruption of the 12/15-lipoxygenase gene diminishes atherosclerosis in apo E–deficient mice*.* J Clin Invest 1999: 103: 1597-1604.

s7. Hatley M E, S Srinivasan, K B Reilly *et al.* Increased Production of 12/15 Lipoxygenase Eicosanoids Accelerates Monocyte/Endothelial Interactions in Diabetic db/db Mice*.* J Biol Chem 2003: 278: 25369-25375.

S8. Shreedhar V, T Giese, V W Sung *et al.* A cytokine cascade including prostaglandin E2, IL-4, and IL-10 is responsible for UV-induced systemic immune suppression*.* J Immunol 1998: 160: 3783-3789.

S9. Schornagel I J, V Sigurdsson, E H Nijhuis *et al.* Decreased neutrophil skin infiltration after UVB exposure in patients with polymorphous light eruption*.* J Invest Dermatol 2004: 123: 202-206.
